# Impact of anatomic origin of primary squamous cell carcinomas of the nasal cavity and ethmoidal sinus on clinical outcome

**DOI:** 10.1007/s00405-018-5068-3

**Published:** 2018-07-19

**Authors:** Stefan Janik, Mariel Gramberger, Lorenz Kadletz, Johannes Pammer, Matthaeus Ch. Grasl, Boban M. Erovic

**Affiliations:** 1 Department of Otorhinolaryngology, Head and Neck Surgery, Medical University Vienna, Vienna, Austria; 2 Clinical Institute of Pathology, Medical University Vienna, Vienna, Austria; 3 Institute of Head and Neck Diseases, Evangelical Hospital Vienna, Hans-Sachs Gasse 10-12, 1180 Vienna, Austria

**Keywords:** Nasoethmoidal carcinoma, Nasal carcinoma, Nodal involvement, Anatomic subsite, Outcome, Elective neck dissection

## Abstract

**Purpose:**

Since squamous cell carcinomas (SCCs) of the nasoethmoidal complex are rare and aggressive malignancies, the purpose of this study was to evaluate whether anatomic subsites of SCCs of the nasal cavity and ethmoid sinuses affect clinical outcome.

**Methods:**

We retrospectively analyzed data from 47 patients with primary SCCs of the nasal cavity and ethmoid sinuses who were treated at the Department of Otorhinolaryngology, Head and Neck Surgery, Medical University of Vienna, between 1993 and 2018. The impact of anatomic subsites of nasoethmoidal SCCs was evaluated with respect to tumor and nodal classification, disease-free survival (DFS) and disease-specific survival (DSS).

**Results:**

Of the 47 cases, 17 SCCs (36.2%) originated from lateral nasal wall followed by 13 (27.7%) tumors of the edge of naris to mucocutaneous junction, 11 (23.4%) SCCs of the nasal septum, 3 tumors of the nasal floor (6.4%) and 3 SCCs of the ethmoid sinuses (6.4%), respectively. SCCs of the nasal septum were associated with significantly higher rates of neck node metastasis (*p* = 0.007), which represented a significantly worse prognostic factor for DSS (HR 7.87; *p* < 0.001). Moreover, advanced tumor stage (HR 5.38; *p* = 0.014) and tumor origin of nasal septum (HR 4.05; *p* = 0.025) were also significantly worse prognostic factors for DSS. Fourteen patients (29.8%) developed recurrent disease, including eight local (17.0%), five regional (10.6%) and one distant (2.1%) recurrence. Elective neck dissection (ND) was associated with lower (0 vs. 20.0%) but not significantly different regional and distant DFS (*p* = 0.075).

**Conclusion:**

Anatomic origin of nasal SCC has significant impact on clinical outcome. SCCs of the nasal septum were associated with higher rates of positive neck nodes and worse outcome.

## Introduction

Carcinomas of the nasal cavity and paranasal sinuses represent orphan malignancies that account for less than 3% of head and neck tumors [1]. The vast majority of tumors are squamous cell carcinomas (SCCs) [1]. Patients with carcinomas of the nasal cavity typically develop early symptoms, including epistaxis or pain, fostering early diagnosis and therapy,  in contrast to carcinomas of the paranasal sinuses [1].

Due to aggressive local spread, rapid growth and tendency for local and regional recurrence, management of sinonasal SCCs is challenging [2, 3]. Radical tumor resection followed by either adjuvant radiotherapy or observation represents the mainstay of therapy [4]. Particularly, endoscopic approaches are increasingly used in treatment of nasal cavity SCCs beside traditional open approaches, such as lateral rhinectomy or degloving facial approaches [5].

Five-year disease specific survival (DSS) ranges from 50 to 80% [6–11]. Nodal involvement, advanced tumor stage and larger tumor size are worse prognostic factors for outcome [1, 2]. Nodal involvement represents a strong worse prognostic factor that results in almost a halving of survival [6–11]. A recently published meta-analysis among 1283 patients with sinonasal carcinomas revealed that elective neck treatment could reduce the risk of regional recurrence [2]. Despite that, the prognostic impact of elective neck treatment or neck dissection (ND) in nasoethmoidal SCCs has not yet been fully elucidated.

Moreover, the impact of human papilloma virus (HPV) infection on outcome in nasal SCCs has been evaluated. First results indicate that HPV positivity seems to represent a favorable prognostic factor for survival [12, 13], while histologic grade does not [11].

Sinonasal carcinomas are differentiated according to the American Joint Committee on Cancer (AJCC), into tumors arising from the nasal cavity and ethmoid sinuses (nasoethmoidal complex) or tumors arising from the maxillary sinuses [14]. Turner JH et al. could show that survival and outcome of patients with sinonasal cancer significantly differ according to histology and tumor site [1]. While it is already known that the location and extend of lesion within maxillary sinus has prognostic significance [14], it is unknown whether anatomic subsites of nasoethmoidal SCCs have also impact on clinical outcome.

Therefore, it was the primary aim of the study to assess the impact of anatomic subsites on outcome in patients with SCCs of the nasal cavity and ethmoid sinuses. Additional information regarding association of anatomic subsite and tumor behavior might be helpful to identify those patients with increased risk for nodal involvement and worse outcome. As a consequence, initial treatment, including elective nodal therapy and oncologic follow-up could be optimized to improve outcome of these patients.

## Materials and methods

### Study population

This retrospective study was conducted at the Department of Otorhinolaryngology, Head and Neck Surgery, Medical University of Vienna. We identified 57 patients with SCCs of the nasoethmoidal complex who were treated between 1993 and 2018. We excluded patients with carcinomas of the maxillary sinuses, nasal vestibule and those with primary carcinomas of the skin. Furthermore, 10 out of these 57 patients with SCCs of the nasoethmoidal complex were excluded due to  recurrent and primary disease. Hence, data of 47 patients with primary SCCs of the nasal cavity and ethmoid sinuses were available for analysis. Hospital medical records were used to obtain information regarding anatomic subsite, clinical characteristics, including symptoms, staging, tumor classification, histologic grading, p16 status, treatment, and outcome. Approval was obtained from the ethics committee of the Medical University Vienna (2329/2016).

### Tumor characteristics

The most recent TNM staging system (8th edition) of the AJCC was used for clinical staging of nasal carcinomas [14]. Moreover, we differentiated nasoethmoidal SCCs according to their anatomic subsites, into malignancies originating from the lateral nasal wall, nasal septum, nasal floor, edge of naris to mucocutaneous junction and ethmoid sinus [14].

Additionally, p16 status, which represents an established surrogate marker for HPV infection in head and neck SCCs, was available and assessed in 35 out of 47 patients (74.5%) [15]. Immunohistochemical staining of p16 was routinely performed by the Clinical Institute of Pathology according to standardized protocols provided by Ventana (CINtec® p16 Histology Kit) [16]. Cytoplasmic staining intensity of tumor cells was calculated as 0 (none), weak (1), moderate (2) or strong (3). Specimens were only counted as positive if more than 90% of tumor cells showed strong (3) p16 expression.

### Outcome analysis

We used disease free survival (DFS) and disease specific survival (DSS) as main outcome parameters for this study. DFS was calculated from date of surgery to date of recurrence or date of last follow-up. Conversely, DSS was calculated from date of surgery to the date of death from nasoethmoidal SCC or date of last follow-up. Unrelated deaths or unknown causes of death were censored.

### Statistical methods

Statistical analyses were performed using SPSS software (version 22; IBM SPSS Inc., IL, USA). Descriptive statistics were used for analysis of demographic and clinical data, tumor characteristics, including histology, tumor classification and staging, anatomic subsites, therapy and outcome. Chi-square test was used to assess associations between nominal variables, such as lymph node classification and anatomic subsites. Additionally, unpaired Student’s *t* test was used to analyze means of normally distributed variables of two independent groups. Kaplan–Meier analysis and log-rank test were performed to determine the impact of different clinical variables on DFS and DSS. Univariable  Cox-regression analyses were calculated to assess the prognostic value of following variables on DFS and DSS: T-classification (T1–T2 vs. T3–T4), N-classification (N neg. vs. N pos.), staging (stage I–II vs. III–IV), anatomic subsite (nasal septum vs. other), p16 status (pos. vs. neg.) and elective ND (yes vs. no). Due to the small patient number, multivariable  Cox-regression analyses were not performed. Hazard ratios (HRs) and 95% confidence intervals (95% CI) are indicated. All tests were two-sided and *p* values below 0.05 were considered as statistically significant. Data are indicated as mean ± standard deviation (SD) within result section. GraphPad Prism 7 (GraphPad Software Inc., California, USA) was used for graphical display of all box plots and Kaplan–Meier curves in this manuscript.

## Results

### Clinical data

For this study, we recruited a total of 47 patients, including 17 (36.2%) females and 30 (63.8%) males, with a mean age of 61.1 ± 14.2 years. All patients had primary SCCs of the nasal cavity or ethmoid sinuses.

Among them, 41 (87.2%) patients suffered from symptoms, while 6 (12.8%) did not. Epistaxis (27.7%) and pain (27.7%) were reported as leading symptoms followed by swelling (17.0%), foreign body sensation (8.5%) and nasal obstruction (6.4%). Median time between first occurrence of symptoms and diagnosis was 4.5 months (range 1–300 months). CT-scan and MRI were performed in 39 (83.0%) and 27 (57.4%) patients for clinical staging. Biopsy was performed in 45 out of 47 patients (95.7%) for diagnostic purpose and histologic evaluation, while in two patients small tumors were resected without previous biopsy.

Within our cohort, there were 30 smokers (63.8%), 10 patients with less (21.3%) and 20 patients (42.6%) with more than 20 pack/years. Additionally, 12 patients (25.5%) had already developed malignant disease, including four basal cell carcinomas, two colon carcinomas, two cervical cancers, two laryngeal carcinomas, one oropharyngeal carcinoma, one multiple myeloma and one melanoma (Table 1).

**Table 1:** Clinical characteristics

Clinical characteristics	Nr.	%
**Sex**		
Male	30	63.8
Female	17	36.2
**Age, years (mean ± SD)**	61.1 ± 14.2	
**Symptoms**		
No symptoms	6	12.8
Symptoms	41	87.2
Epistaxis	13	27.7
Pain	13	27.7
Swelling	8	17.0
Foreign body sensation	4	8.5
Nasal obstruction	3	6.4
**Duration of symptoms, months (median ± SD)**	4.5 ± 49.0	
**Biopsy**		
No	2	4.3
Yes	45	95.7
**Staging**		
Yes	47	100.0
CT	39	83.0
MRI	27	57.4
**Smoking history**		
Never	17	36.2
< 20 pack-years	10	21.3
> 20 pack-years	20	42.6
**Previous malignancies**		
Yes	12	25.5
No	35	74.5

*SD* Standard deviation, *Nr*. number of patients

### Tumor origin and tumor behavior

According to the 8th edition of the AJCC staging system, we had 20 patients with T1 (42.6%), 14 patients with T2 (29.8%), 6 patients with T3 (12.8%), 6 patients with T4a (12.8%) and 1 patient with T4b (2.1%) tumors. Mean tumor size was 2.3 ± 1.5 cm. At time of diagnosis, six patients (12.8%) presented with positive neck nodes, from which one had N1 (2.1%), three had N2b (6.4%) and two had N2c (3.0%) disease, respectively. Only one patient (2.1%) presented already with bone metastases in vertebral column (M1). Hence, we had 19 stage I (40.4%), 11 stage II (23.4%), 5 stage III (10.6%) and 12 stage IV (25.5%) patients (Table 2).

**Table 2:** Tumor characteristics

Tumor characteristics	Nr.	%
**T classification**		
T1	20	42.6
T2	14	29.8
T3	6	12.8
T4a	6	12.8
T4b	1	2.1
**N-classification**		
N0	41	87.2
N1	1	2.1
N2	5	10.7
N2a	0	0
N2b	3	6.4
N2c	2	4.3
N3	0	0
**M classification**		
M0	46	97.9
M1	1	2.1
**Tumor stage**		
Stage I	19	40.4
Stage II	11	23.4
Stage III	5	10.6
Stage IV	12	25.5
**Anatomic subsites**		
Lateral wall	17	36.2
Edge of naris to mucocutaneous junction	13	27.7
Nasal septum	11	23.4
Floor	3	6.4
Ethmoid sinus	3	6.4
**Grading**		
G1 (well differentiated)	7	14.9
G2 (moderately differentiated)	29	61.7
G3 (poorly differentiated)	11	23.4
**p16 status**		
Negative	27	57.4
Positive	8	17.0
Unknown	12	25.5

*Nr*. Number of patients, *T* tumor classification, *N* lymph node status, *M* presence of metastasis

**Fig. 1:**
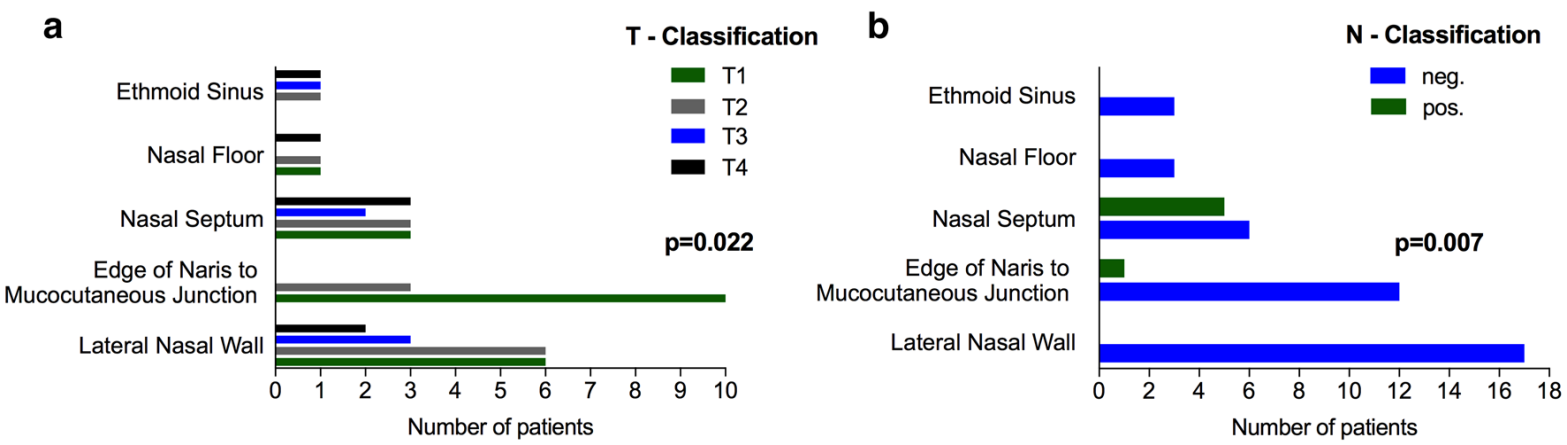
Influence of anatomic subsite on T- and N-classification. T-classification significantly correlates with tumor origin (*p* = 0.022). T4a (*n* = 6) and T4b (*n* = 1) tumors have been summarized as T4 tumors (**a**). Furthermore, 5 out of 11 patients with carcinomas of the nasal septum presented with positive neck nodes, while positive lymph nodes were rare or absent in carcinomas of other anatomic subsites (*p* = 0.007; **b**)

The majority of tumors originated from lateral nasal wall (36.2%) followed by tumors of the edge of naris to mucocutaneous junction (27.7%), nasal septum (23.4%), nasal floor (6.4%) and ethmoid sinuses (6.4%) (Table 2). T-classification significantly differ according to anatomic subsite (*p* = 0.022; Fig. 1a). In particular, tumor originating from the edge of naris to mucocutaneous junction, lateral nasal wall and nasal floor presented in 100%, 70.6% and 66.7% with T1 and T2 SCCs compared to 54.5% and 33.3% T1 and T2 tumors in SCCs of the nasal septum and ethmoid sinuses, respectively.

Moreover, 5 out of 11 SCCs that originated from the nasal septum (45.5%) presented with positive neck nodes, while initial neck node involvement was rare in SCCs of other anatomic subsites (*p* = 0.007; Fig. 1b). Except tumor origin, N-classification was not significantly influenced by T-classification (T1–T2 vs. T3–T4; *p* = 0.639), tumor size (*p* = 0.880), tumor grading (*p* = 0.511) or p16 status (*p* = 0.189), respectively.

### Tumor grading and p16 status

**Fig. 2:**
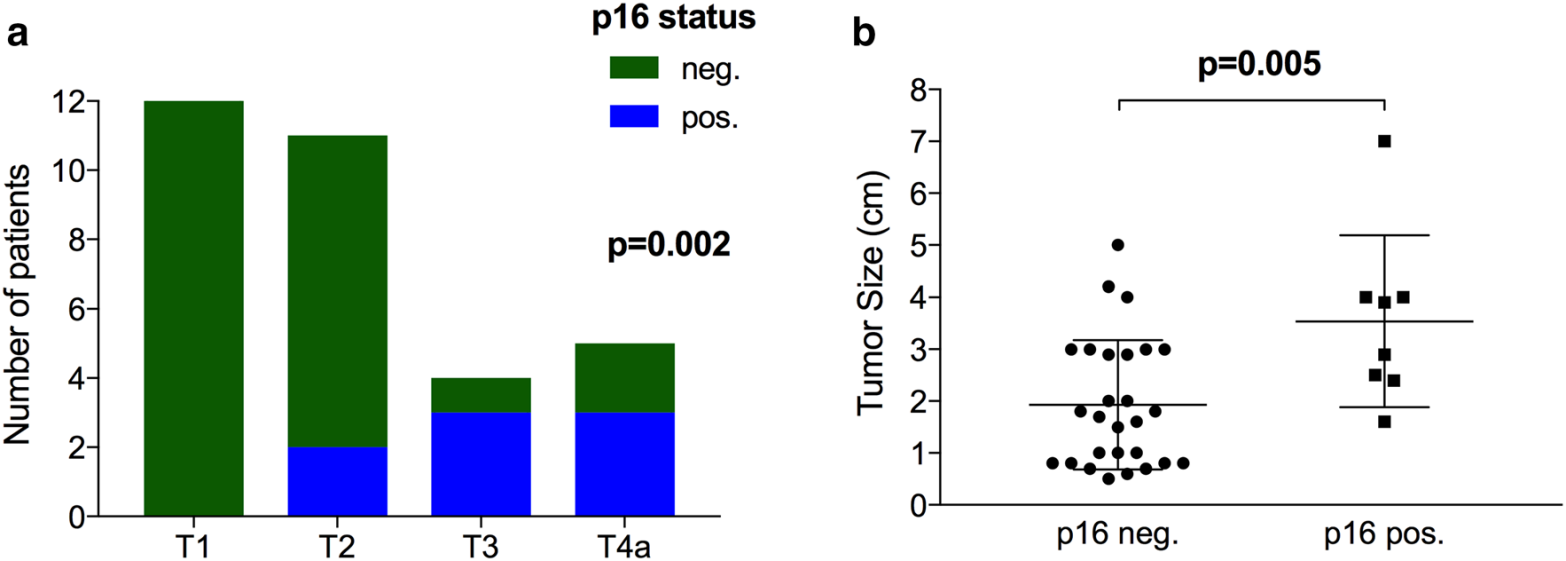
Tumor classification and tumor size correlates with p16 status. Positive p16 expression was missing in T1 tumors, but was found in 75.0% and 60.0% of T3 and T4a carcinomas (*p* = 0.002; **a**). Moreover, mean tumor size was significantly higher in p16 positive compared to negative tumors (*p* = 0.005; **b**)

Regarding tumor grading, 61.7% of SCCs were moderately differentiated (G2), 23.4% were poorly differentiated (G3) and 14.9% showed well differentiated (G1) morphology. Information of p16 status was available in 35 patients (74.5%). Among them, positive and negative p16 expression was found in 17.0% (*n* = 8) and 57.4% (*n* = 27) of patients, and  p16 status significantly corresponds to T-classification (*p* = 0.002) (Table 2). In particular, 0%, 18.2%, 75.0% and 60% of T1, T2, T3 and T4a tumors were p16 positive (Fig. 2a), respectively. Furthermore, mean tumor size was 3.5 ± 1.7 cm in p16 positive SCCs, which was significantly larger compared to p16 negative tumors with a mean tumor size of 1.9 ± 1.2 cm (*p* = 0.005; Fig. 2b).

### Treatment and neck dissection

Surgical tumor resection alone was done in 68.1% of cases (*n* = 32), while 21.3% underwent multimodal treatment regimes combining surgery with radiotherapy (RT) and/or chemotherapy (*n* = 10). Radical tumor resection (R0) was achieved in 38 out of 42 patients (90.5%). The remaining five patients were treated with primary radiochemotherapy (RChT). Three patients with T4a nasoethmoidal SCCs disclaimed extensive surgery with exenteration and free-flap reconstruction, while one patient with T4b SCCs (infiltration of skull base) and one patient with distant metastases were no candidates for curative surgery. Unilateral and bilateral ND was performed in 4 (8.5%) and 11 patients (23.4%), respectively. Positive lymph nodes were detected in 4 of these 15 neck-dissected patients (26.7%). Subsequently, elective ND was performed in 11 out of 41 patients (26.8%).

### Survival analysis

Mean and median follow-up was 36.9 and 33.7 months (range 0.1–227.2 months), respectively. Of the 47 patients, 14 (29.8%) experienced tumor recurrence, including 8 local (17.0%), 5 regional (10.6%) and 1 distant (2.1%) recurrence. We performed Kaplan–Meier survival analyses to determine if DFS and DSS were influenced by T-classification (T1–T2 vs. T3–T4), N-classification (N neg. vs. N pos.), tumor stage (stage I–II vs. III–IV), anatomic subsite (nasal septum vs. other), p16 status (pos. vs. neg.) and elective ND (yes vs. no).

DFS was not significantly affected by one of the tested variables (Table 3). Nevertheless, it is noteworthy to mention that none of those patients who underwent elective ND experienced regional or distant tumor recurrence. In particular, 5-year regional and distant DFS was 100% in patients who underwent elective ND compared to 68% in those who did not, but this difference failed to reach statistical significance (*p* = 0.075).

**Table 3:** Kaplan–Meier survival analyses

Characteristics		Nr.		Disease free survival (DFS)				Disease specific survival (DSS)			
				1 year	3 years	5 years	*p*	1 year	3 years	5 years	*p*
**T-classification**											
T1–T2		35		73.8	65.1	65.1		96.4	79.4	70.0	
T3–T4		12		57.1	57.1	57.1	0.381	90.0	80.0	53.3	0.420
**N-classification**											
N neg		41		70.0	62.6	62.6		100.0	89.3	76.5	
N pos		6		66.7	66.7	66.7	0.848	66.7	33.3	16.7	< 0.001
**Tumor stage**											
Stage I–II		30		73.3	63.5	63.5		100.0	94.4	82.6	
Stage III–IV		17		63.5	63.5	63.5	0.628	87.1	60.3	43.0	0.006
**Anatomic subsite**											
Nasal septum		11		60.0	60.0	60.0		90.0	35.0	35.0	
Other^a^		36		72.3	64.0	64.0	0.536	96.6	88.7	75.4	0.016
**p16 status**											
pos		8		50.0	25.0	25.0		100.0	100.0	66.7	
neg		27		71.0	65.5	65.5	0.069	90.9	75.1	69.8	0.607
Elective ND^b^											
Yes		11		100.0	100.0	100.0		100.0	90.0	90.0	
No		30		84.2	68.0	68.0	0.075	100.0	89.2	68.6	0.269

*Nr*. Number of patients, *p p* value

^a^Other: tumors originating from other anatomic subsites than nasal septum

^b^The impact of elective neck dissection (ND) was only evaluated in NO diseases (*n* = 41) for regional and distant DFS and DSS

**Fig. 3:**
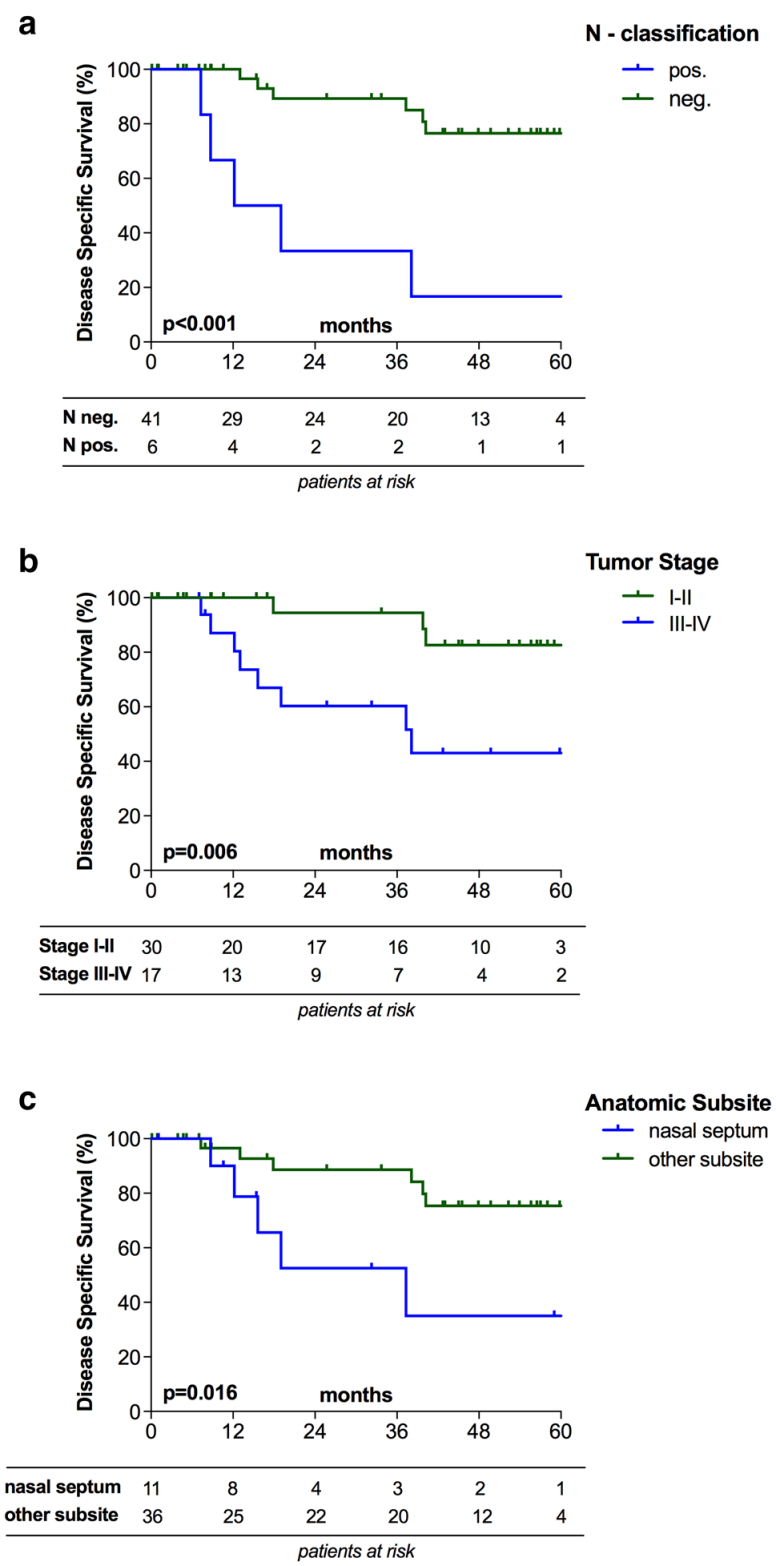
Survival analyses. Positive neck nodes (N pos.), advanced tumor stage (stage III–IV) and tumor origin of nasal septum were associated with significantly worse disease specific survival (**a**, **b**, **c**)

Moreover, patients with positive neck nodes (N pos.), stage III–IV nasoethmoidal SCCs and tumors originating from nasal septum showed significantly worse DSS (*p* < 0.001; *p* = 0.006 and *p* = 0.016; respectively; Fig. 3). Conversely, T-classification, p16 status and elective ND did not significantly influence DSS (*p* = 0.420; *p* = 0.607 and *p* = 0.269) (Table 3).

### Prognostic factors

Finally, we performed Cox-regression analyses to assess the prognostic power of the abovementioned clinical variables on DFS and DSS. While none of the tested variables significantly impacted DFS, positive neck nodes (HR 7.87; *p* = 0.001), advanced tumor stage (stage III–IV; HR 5.38; *p* = 0.014) and tumor origin of nasal septum (HR 4.05; *p* = 0.025) were significantly worse prognostic factors for DSS at univariable analysis (Table 4).

**Table 4:** Univariable Cox—regression analysis

	Univariable analysis		
	HR	*p* value	95% CI
**Disease free survival**			
T-classification (T3–T4 vs. T1–T2)	1.62	0.386	0.54–4.85
N-classification (N pos. vs. N neg.)	1.16	0.849	0.26–5.18
Tumor stage (III–IV vs. I–II)	1.30	0.629	0.45–3.78
Anatomic subsite (septum vs. other^a^)	1.46	0.538	0.45–4.61
p16 status (pos. vs. neg.)	2.79	0.081	0.88–8.85
Elective ND (no vs. yes)^b^	45.9	0.319	0.03–100.0
**Disease specific survival**			
T-classification (T3–T4 vs. T1–T2)	1.65	0.425	0.48–5.68
N-classification (N pos. vs. N neg.)	7.87	0.001	2.35–26.3
Tumor stage (III–IV vs. I–II)	5.38	0.014	1.41–20.4
Anatomic subsite (septum vs. other^a^)	4.05	0.025	1.20–13.7
p16 status (pos. vs. neg.)	0.58	0.628	0.23–4.81
Elective ND (no vs. yes)^b^	3.15	0.295	0.37–27.0

*HR* Hazard ration, *95% CI* 95% confidence interval

^a^Other: tumors originating from other anatomic subsites than nasal septum, including lateral nasal wall, nasal floor, edge of naris to mucocutaneous junction and ethmoid sinuses

^b^Elective neck dissection (ND) was only done in patients with cN0 disease. Therefore regional and distant disease free survival and disease specific survival was only calculated in a subset of 41 patients, while the other variables were tested for the whole cohort of 47 patients

## Discussion

Former studies demonstrated that tumor origin of sinonasal cancers corresponds to clinical stage and outcome [1, 2]. Accordingly, tumors originating of the maxillary sinus are typically associated with higher T-classification and worse prognosis compared to tumors of the nasal cavity. Herein we investigated the importance and impact of anatomical subsites of nasoethmoidal SCCs on clinical outcome.

Within our cohort, SCCs mainly originated from lateral nasal wall followed by tumors of the edge of naris to mucocutaneous junction and nasal septum. Initial nodal involvement was found in 12.8% of our cases, which was similar to previous publications reporting on positive neck nodes in 6.3–14.2% up to 24% of cases [2, 9, 17, 18]. We could show for the first time that anatomical subsite has significant impact on clinical outcome of SCCs of the nasal cavity and ethmoidal sinuses. Particularly, septal carcinomas showed significantly higher rates of lymph node metastases compared to tumors that originated from other anatomic regions of the nasoethmoidal complex (45.5% vs. 2.8%). Patients with positive neck nodes (N pos.) had a seven- to eightfold higher risk to die from nasoethmoidal carcinoma compared to neck node-negative (N neg.) patients. This is in line to former studies demonstrating that nodal involvement represents a significantly worse prognostic factor that cut patients’ overall survival almost to a half compared to node-negative patients [10, 19].

SCCs of the nasal septum were already described as aggressive and often undertreated tumors with high initial nodal involvement rates (6–24%) and early regional neck recurrences in up to 44% of cases, within the first 2 years after initial therapy [7, 18]. To date, the reasons for this aggressive tumor behavior are unclear, but early and frequent lymph node metastatic spread may be linked to the very close and rich lymph capillary network located in particular at the floor of the nose and upper lip [20].

Moreover, anatomic subsite significantly impacts T-stage. Tumors of the anterior nasal cavity, including the lateral nasal wall or edge of naris to mucocutaneous junction, typically cause early symptoms that foster diagnosis and treatment. Subsequently, 100% of tumors of the edge of naris to mucocutaneous junction and 70.6% of tumors of the lateral nasal wall presented with T1 and T2 carcinomas, while 45.5% of septal carcinomas and 66.6% of tumors of the ethmoid sinus were detected as T3 and T4 tumors, respectively.

A recently published SEER (Surveillance, Epidemiology and End Results) analysis evaluated the risk of lymph node metastasis in 1283 patients with sinonasal SCCs [2]. They found out that T4 carcinomas of the nasal cavity and tumor size larger than 2 cm were associated with significantly higher rates of positive lymph nodes, and that lymph nodes at neck levels I and II were most commonly affected [2]. Herein, T4 tumors and larger tumor size were not associated with significantly higher rates of nodal involvement.

Recently, the benefit of elective treatment of N0 disease with radiotherapy or ND has been discussed. In the meta-analysis of Scurry et al., reviewing 23 studies, the risk for regional recurrence was 18.1%, and therefore, they favor elective regional treatment [11]. Conversely, other authors recommend elective treatment only for T4 tumors [10]. However, according to Ahn et al., elective nodal treatment could lead to a five- to sixfold decrease of nodal recurrence in sinonasal carcinomas [2]. In our cohort, elective ND was performed in 11 out of 41 patients (26.8%). Notably, none of these elective neck-dissected patients developed regional or distant recurrence compared to five regional and one distant recurrence in those patients who were not neck dissected. Accordingly, 5-year regional and distant DFS was 100% in patients who underwent elective ND compared to 68% those who did not. Despite this strong trend towards improved regional and distant DFS in patients with nasoethmoidal SCCs and elective ND, this difference did not reach statistical significance, and therefore further prospective studies with larger patient numbers are necessary.

With regards to HPV infection, it has already been shown that HPV infection has a pivotal role for development and prognosis of SCCs of the head and neck. HPV-related oropharyngeal SCCs (OPSCC) represent a different tumor entity compared to HPV-negative OPSCCs. HPV-positive tumors are characterized by advanced clinical stages with smaller primary tumors but higher rates of positive neck nodes. Despite that HPV-positive OPSCCs show significantly better response rates to RT and RChT, better long-time outcome and better loco-regional control. Hence, HPV infection is considered as positive prognostic factor for OPSCC [21–23]. So far, only few data exist regarding the impact of HPV positivity in nasal SCCs. HPV positivity was reported in 10 up to 62% of carcinomas [3, 12] and former works demonstrated that HPV positivity was a favorable prognostic factor for nasal SCCs [12, 13]. Chowdhury et al. showed that median overall survival was 54 months in HPV positive compared to 12 months in HPV negative sinonasal SCCs [12]. Similar to OPSCC, we used p16 staining as surrogate marker for HPV infection. In our cohort, p16 positivity was associated with significantly higher T-stage and significantly higher tumor sizes, which is in contrast to the literature. To the best of our knowledge, this is the first time that p16 positivity was linked to higher T-classification in SCCs of the nasal cavity and ethmoidal sinuses. Conversely to the literature, p16 positivity was no favorable prognostic factor for outcome in our cohort [12, 13].

There are some limitations of our study. The first limitation of our study is the small study population, which is caused by the rarity of nasoethmoidal SCCs. The annual incidence of sinonasal carcinomas is 5.6 cases per million [1]. Subsequently, our study group of 47 patients corresponds to 8.4 million individuals, which is representative for the Austrian population of 8.5 million people. Furthermore, the retrospective study design carries the inherent risk of information bias. Finally, due to unspecific and partially overlapping anatomic boundaries and definitions in nasal cavity, it is sometimes difficult to clearly allocate SCCs to distinct anatomic subsites. As already raised by Becker et al., unselected analysis of carcinomas of the nasal cavity, paranasal sinuses and nasal vestibule, without differentiation according to anatomic subsites, cause inhomogeneous results, which may lead to wrong conclusions [3]. Therefore, all patients with uncertain or incomplete data regarding tumor subsite were excluded from analysis a priori to obtain a homogenous patient cohort.

## Conclusion

Tumor behavior and outcome is strongly influenced and determined by anatomic subsite. Particularly, septal SCCs represented highly aggressive malignancies with higher rates of nodal involvement and subsequently worse outcome. Consequently, high-risk patients, including septal SCCs, may benefit from elective ND or more aggressive treatment regimens. Because we could show that elective ND was associated with better but not significantly different outcome, further studies with higher patient numbers are needed.
